# Bidirectional Causal Connectivity in the Cortico-Limbic-Cerebellar Circuit Related to Structural Alterations in First-Episode, Drug-Naive Somatization Disorder

**DOI:** 10.3389/fpsyt.2018.00162

**Published:** 2018-04-26

**Authors:** Ranran Li, Feng Liu, Qinji Su, Zhikun Zhang, Jin Zhao, Ying Wang, Renrong Wu, Jingping Zhao, Wenbin Guo

**Affiliations:** ^1^Department of Psychiatry, Second Xiangya Hospital of Central South University, Changsha, China; ^2^Department of Radiology, Tianjin Medical University General Hospital, Tianjin, China; ^3^Mental Health Center of the First Affiliated Hospital, Guangxi Medical University, Nanning, China

**Keywords:** somatization disorder, resting-state functional magnetic resonance imaging, voxel-based morphometry, cortico-limbic-cerebellar, gray matter volume, granger causality analysis

## Abstract

**Background:** Anatomical and functional deficits in the cortico-limbic-cerebellar circuit are involved in the neurobiology of somatization disorder (SD). The present study was performed to examine causal connectivity of the cortico-limbic-cerebellar circuit related to structural deficits in first-episode, drug-naive patients with SD at rest.

**Methods:** A total of 25 first-episode, drug-naive patients with SD and 28 healthy controls underwent structural and resting-state functional magnetic resonance imaging. Voxel-based morphometry and Granger causality analysis (GCA) were used to analyze the data.

**Results:** Results showed that patients with SD exhibited decreased gray matter volume (GMV) in the right cerebellum Crus I, and increased GMV in the left anterior cingulate cortex (ACC), right middle frontal gyrus (MFG), and left angular gyrus. Causal connectivity of the cortico-limbic-cerebellar circuit was partly affected by structural alterations in the patients. Patients with SD showed bidirectional cortico-limbic connectivity abnormalities and bidirectional cortico-cerebellar and limbic-cerebellar connectivity abnormalities. The mean GMV of the right MFG was negatively correlated with the scores of the somatization subscale of the symptom checklist-90 and persistent error response of the Wisconsin Card Sorting Test (WCST) in the patients. A negative correlation was observed between increased driving connectivity from the right MFG to the right fusiform gyrus/cerebellum IV, V and the scores of the Eysenck Personality Questionnaire extraversion subscale. The mean GMV of the left ACC was negatively correlated with the WCST number of errors and persistent error response. Negative correlation was found between the causal effect from the left ACC to the right middle temporal gyrus and the scores of WCST number of categories achieved.

**Conclusions:** Our findings show the partial effects of structural alterations on the cortico-limbic-cerebellar circuit in first-episode, drug-naive patients with SD. Correlations are observed between anatomical alterations or causal effects and clinical variables in patients with SD, and bear clinical significance. The present study emphasizes the importance of the cortico-limbic-cerebellar circuit in the neurobiology of SD.

## Introduction

Somatization disorder (SD) is characterized by a history of various unexplained physical symptoms in many organ systems. This disorder begins before age of 30 and occurs for many years, leading to repeated treatment seeking or significant impairment in social/occupational function (1). Lifetime prevalence of SD varies from 0.2 to 2% in women and less than 0.2% in men (2). In the Diagnostic and Statistical Manual of Mental Disorders-IV (DSM-IV), SD is characterized by multiple, recurring, affecting many organ systems, with at least four pain symptoms, two gastrointestinal symptoms, one sexual symptom and one pseudoneurological symptom. Each of the symptoms cannot be explained by a general medical condition, or in factitious disorder or malingering (3). The occurrence of somatization symptoms is commonly assessed by self-report questionnaires. A large amount of questionnaires are available to assess self-report somatization symptoms (4), such as the screen for Somatoform Symptoms (5), Physical Health Questionnaire-15 (6), and somatization subscale of the symptom checklist-90 (SCL-90) (7). Among these questionnaires, the SCL-90 and Physical Health Questionnaire-15 were deemed as the suitable scales for assessing somatization symptoms.

Although evidence suggests that dissociation amnesia, childhood emotional/physical abuse, and unsupportive family environment are associated with SD (8), its neurobiology remains unclear. The functional neuroimaging methods allow us to investigate neural changes in patients with SD (9–14). For instance, Garcia-Campayo et al. (14) reported that patients with SD exhibited hypoperfusion in the frontal, cerebellar, and temporoparietal brain areas under single-photon-emission computed tomography (SPECT). Moreover, increased glutamatergic activity in the posterior cingulate cortex (PCC) was observed in patients with SD by magnetic resonance spectroscopy techniques (9). By contrast, regional cerebral hypometabolism in the right precentral gyrus, caudate nuclei, and left putamen was found in patients with SD by using positron emission tomography techniques (15). Recently, abnormal activities in the anterior ventral precuneus, PCC, and anteromedial thalamus were correlated with the somatization severity of SD (16). Patients with SD showed increased regional activity in the bilateral superior medial prefrontal cortex (MPFC) and decreased regional activity in the left precuneus (12). The same researchers also found a positive correlation between increased activity in the bilateral superior MPFC and the scores of SCL-90 (12), and an increase in the right inferior temporal gyrus functional connectivity (FC) in patients with SD (13). Moreover, a variety of literatures reported correlations between abnormal FC or neural activity within brain areas of the default-mode network (DMN) and somatization severity or personality (10, 11, 17, 18). Impaired brain activity has been shown in patients with SD under an emotional empathy task by using functional magnetic resonance imaging (fMRI) (19), such as reduced activity in the bilateral parahippocampal gyrus, left amygdala, and left superior temporal gyrus, suggesting that these brain regions are responsible for emotional regulation and emotional memory.

Limited structural imaging studies have shown that patients with SD exhibit anatomical alterations, including reduced pituitary and amygdala volume and increased caudate nucleus volume (20–22). By contrast, no significant white matter differences were found between patients with SD and healthy controls by using diffusion tensor imaging at the corrected level (23). However, the same study showed that patients with SD had significantly decreased fractional anisotropy values in the right cingulate cortex and right inferior fronto-occipital fasciculus compared with controls at the uncorrected level in the same study (23), and the fractional anisotropy values of the two brain regions are correlated with the severity of somatization symptoms.

Furthermore, cerebellar alterations were observed in patients with SD, such as increased cerebellar-DMN FC (10) and decreased regional homogeneity in the left cerebellum (24). In addition, a recent study found that patients with persistent somatoform pain disorder showed increased FC between the sensorimotor network and cerebellar network (25). In a study comparing empathic deficits in patients with somatoform disorder using fMRI during an empathy task, de Greck et al. (19) observed lower activity in several brain regions, such as the bilateral parahippocampal gyrus, left amygdala, left superior temporal gyrus, left postcentral gyrus, bilateral cerebellum, and left posterior insula.

The aforementioned studies revealed that the cortico-limbic-cerebellar circuit may play a crucial role in the neurobiology of the SD. However, several methodological drawbacks should be taken into consideration when interpreting these findings. First, some studies have used structural MRI for data analysis without combining with fMRI to analyze neural activity or FC in patients with SD, thus preventing the understanding of the relationship between anatomical and functional alterations. Second, most studies have adopted regions of interest (ROI) or independent component analysis (ICA). The reported results might be affected by the selection of ROIs or uncertainty of ICA signal separation. Importantly, the altered information flow of the cortico-limbic-cerebellar circuit remains unclear based on prior studies.

**Figure 1:**
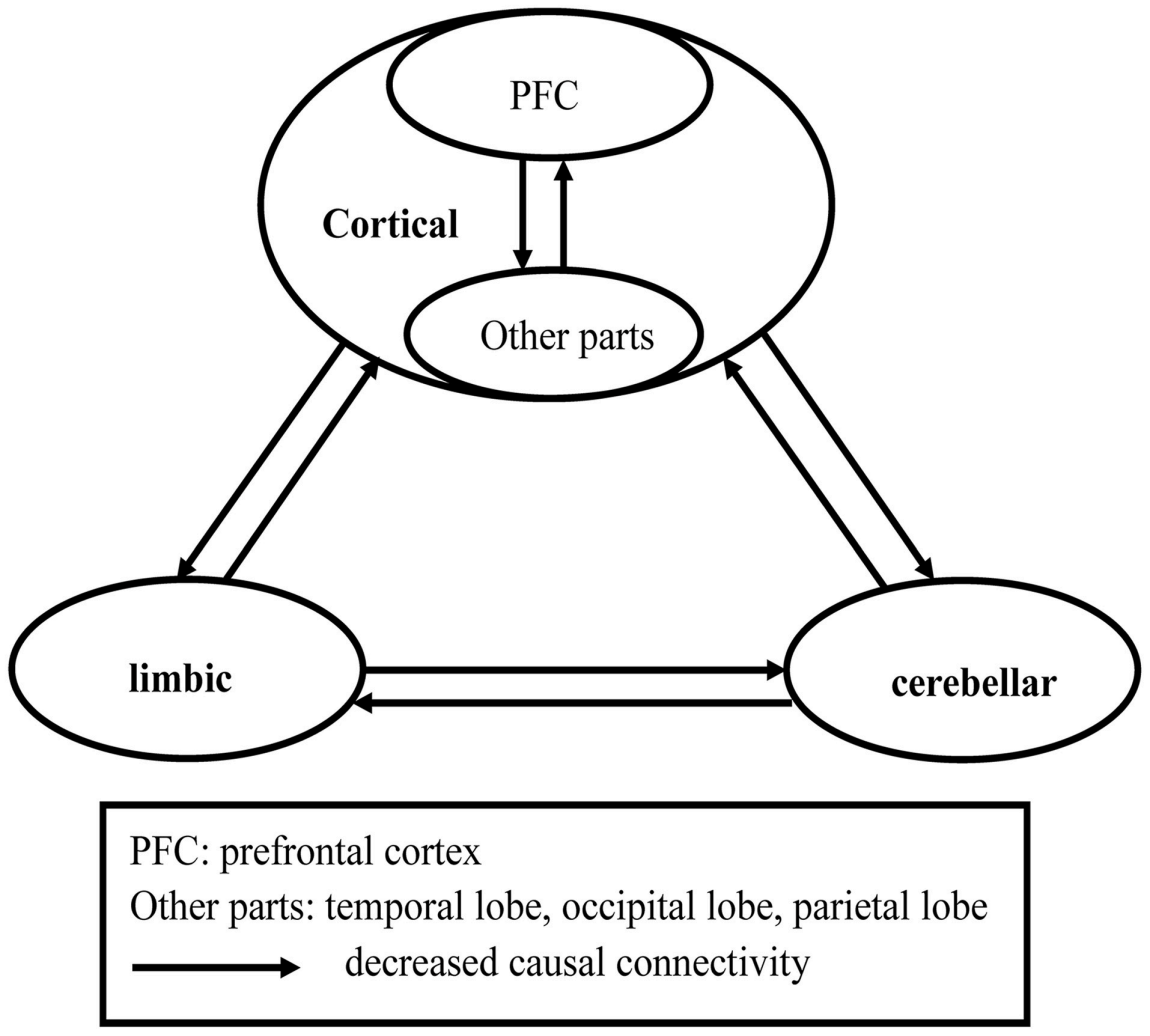
Hypothesized model in first-episode, drug-naive somatization disorder: overall reduced causal connectivities in the cortico-limbic-cerebellar circuit.

In the present study, we used voxel-based morphometry (VBM) to examine whole-brain GMV differences in patients with SD, and brain areas with abnormal GMV were selected as seeds. Then, Granger causality analysis (GCA) was employed to examine abnormal causal connectivity of the seeds with other voxels of the entire brain. The GCA method is based on the predictive value of the current time series Y from the past value of time series X for reasoning that the causal influence from X causes Y (26). The Granger effect was assessed by a signed regression coefficient β (27, 28). Here, we aimed to determine the anatomical deficits and causal connectivity related to anatomical deficits in a group of first-episode, drug-naive patients with SD. Based on the abovementioned researches, we hypothesized that patients with SD would show anatomical deficits of the cortico-limbic-cerebellar circuit, and causal effects would decrease with the anatomical deficits (see Figure 1). We also examined the correlations between the abnormal GMV or causal connectivity, and clinical variables (i.e., symptom severity and cognitive function) in the patients.

## Materials and methods

### Participants

A total of 56 right-handed subjects were recruited for this study, including 26 first-episode, drug-naive patients with SD and 30 healthy controls. Diagnosis of SD was determined through a consensus of two experienced clinical psychiatrists by using the Structural Clinical Interview of the Diagnostic and Statistical Manual of Mental Disorders-IV (SCID), Patient's Edition (29). Healthy controls were recruited from the community and were screened to exclude lifetime psychiatric illness by the non-patient edition of the SCID. Moreover, healthy controls with a history of psychiatric illness in first-degree relatives were also excluded. Exclusion criteria for all subjects included severe medical or neurological diseases, substance abuse or loss of consciousness history, mental retardation, contraindications for MRI scanning, and other psychiatric disorders, such as schizophrenia, bipolar disorder, anxiety disorders, and personality disorders. As depressive symptoms were common in patients with SD, patients with comorbidity of depression were not excluded. However, the presence of depressive symptoms should occur after the onset of somatization symptoms. Six patients with SD presented comorbidity with major depressive disorder in the current study. In addition, none of the healthy controls had a personal history of psychiatric or severe physical disease and craniocerebral operations.

All subjects were evaluated through the following tests by two experienced psychiatrists (ZZ and WG): symptom severity of somatization, depression, and anxiety were assessed by the somatization subscale of SCL-90 (7), Hamilton Rating Scale for Depression (HAMD, 17 items) (30), and Hamilton Anxiety Scale (HAMA) (31); Eysenck Personality Questionnaire (EPQ) (32) was used to assess personality dimensions; and cognitive function was assessed by the Wisconsin Card Sorting Test (WCST) (33) and Wechsler Adult Intelligence Scale (WAIS): digit symbol coding (34).

This study was carried out in accordance with the recommendations of the local ethics committee of the First Affiliated Hospital of Guangxi Medical University. All subjects gave a written informed consent in accordance with the Declaration of Helsinki.

### MRI acquisition and functional data preprocessing

Whole-brain imaging was acquired on a 3.0 T Siemens scanner. Functional data were preprocessed with the software DPABI in Matlab (35). Details of MRI acquisition and functional data preprocessing are provided in the Supplementary Files.

### Anatomical analyses

Each image was manually checked for gross anatomical abnormalities and image artifacts. The images were processed with the VBM8 toolbox (http://dbm.neuro.uni-jena.de/vbm) and SPM8 (http://www.fil.ion.ucl.ac.uk/spm). First, the images were normalized to the same template using a 12 parameter affine transformation. Afterward, each participant's images were segmented to identify tissue-signal intensities, combined with prior knowledge of probability maps. The images were then spatially normalized to the template space and resampled to 1.5 × 1.5 × 1.5 mm^3^. To eliminate non-brain tissue voxels from dural venous sinuses, skull, scalp, and diplopic space, an automated brain extraction procedure was used. Finally, the optimally normalized segmented images were modulated, and the obtained images were smoothed with an 8 mm full-width half-maximum Gaussian kernel.

Two-sample *t*-tests were conducted to determine GMV differences between patients with SD and healthy controls. Age was used as a covariate to minimize the potential effects of this variable. For multiple comparisons, the significance threshold was set at *p* < 0.05 for multiple comparisons corrected by the Gaussian Random Field (GRF) theory (voxel significance: *p* < 0.001, cluster significance: *p* < 0.05).

### GCA processing

Four brain regions, the right cerebellum Crus I, right middle frontal gyrus (MFG), left anterior cingulate cortex (ACC), and left angular gyrus (AG), with abnormal GMV were selected as seeds. The peak voxel of each seed was selected as a 6 mm-radius spherical seed for GCA processing. Voxel-wise coefficient GCA was performed by using the REST software (36). Granger causality was conducted by vector autoregressive models to explore whether the past variable of a time series could predict the current variable of another time series correctly. A signed regression coefficient β was used to estimate the Granger effect (27, 28). Positive/negative β may indicate an excitatory/inhibitory effect or positive/negative feedback (37). There were two analyses for each seed: seed-to-whole-brain and whole-brain-to-seed analyses. The former was conducted to estimate the driving effect from the seed to other brain regions of the whole brain, including excitatory and inhibitory effects. The latter was used to estimate the feedback effect from other brain regions of the whole brain to the seed, including positive and negative feedback. Two sample *t*-tests were used to compare the causal effects between patients and controls. The framewise displacement (FD) values were computed for all subjects. Age and the mean FD values were used as covariates in the group comparisons to minimize the potential effects of these variables. The significance threshold was set at *p* < 0.05 (GRF corrected).

### Correlation analyses

To identify the correlations between abnormal GMV or causal effect and symptoms in patients with SD, partial correlation analyses were conducted after controlling for the HAMD and HAMA scores to rule out the potential effects of depression and anxiety. The statistical threshold was set at *p* < 0.05 (Bonferroni corrected).

## Results

### Demographics and clinical characteristics of participants

The data of one patient and two controls were eliminated due to excessive head motion. As shown in Table 1, no significant differences were found between patients and controls in regard to age, sex ratio, education level, digit symbol coding of WAIS, EPQ extraversion and EPQ lie scores, and WCST scores. Relative to healthy controls, patients with SD showed significantly higher scores in the SCL-90 somatization subscale, HAMD, HAMA, and EPQ psychoticism and neuroticism scales. Furthermore, the controls exhibited higher FD values than those of the patients.

**Table 1:** Characteristics of the participants.

**Variables**	**Patients (*n* = 25)**	**Controls (*n* = 28)**	***p*-value**
Age (years)	41.00 ± 10.76	38.71 ± 9.59	0.42^b^
Sex (male/female)	4/21	6/22	0.73^a^
Years of education (years)	7.72 ± 4.39	7.82 ± 2.59	0.92^b^
FD (mm)	0.08 ± 0.03	0.10 ± 0.05	0.02^b^
Illness duration (months)	59.12 ± 62.22		
Somatization subscale of SCL-90	28.48 ± 10.37	14.32 ± 3.44	<0.001^b^
HAMD	18.84 ± 7.31	2.60 ± 1.83	<0.001^b^
HAMA	22.96 ± 10.95	0.53 ± 0.99	<0.001^b^
Digit symbol-coding of WAIS	8.28 ± 2.87	9.64 ± 2.15	0.06^b^
EPQ			
Extraversion	46.84 ± 11.02	49.75 ± 9.65	0.31^b^
Psychoticism	50.52 ± 9.01	45.00 ± 8.54	0.03^b^
Neuroticism	57.36 ± 9.18	46.78 ± 10.24	<0.001^b^
Lie	49.44 ± 12.31	47.96 ± 11.01	0.65^b^
WCST			
Number of categories achieved	3.52 ± 1.76	3.89 ± 1.66	0.43^b^
Number of errors	22.84 ± 9.12	24.71 ± 8.91	0.45^b^
WCST-Pre	20.04 ± 9.48	22.82 ± 8.72	0.27^b^

a *The p-value for sex distribution was obtained by a chi-square test*.

b *The p-values were obtained by two sample t-tests*.

*FD, Framewise displacement; HAMD, Hamilton depression scale; HAMA, Hamilton anxiety scale; SCL-90, Symptom Checklist-90; EPQ, Eysenck Personality Questionnaire; WAIS, Wechsler Adult Intelligence Scale; WCST-Pre, persistent error response of Wisconsin Card Sorting Test*.

### Anatomical abnormalities between groups

**Figure 2:**
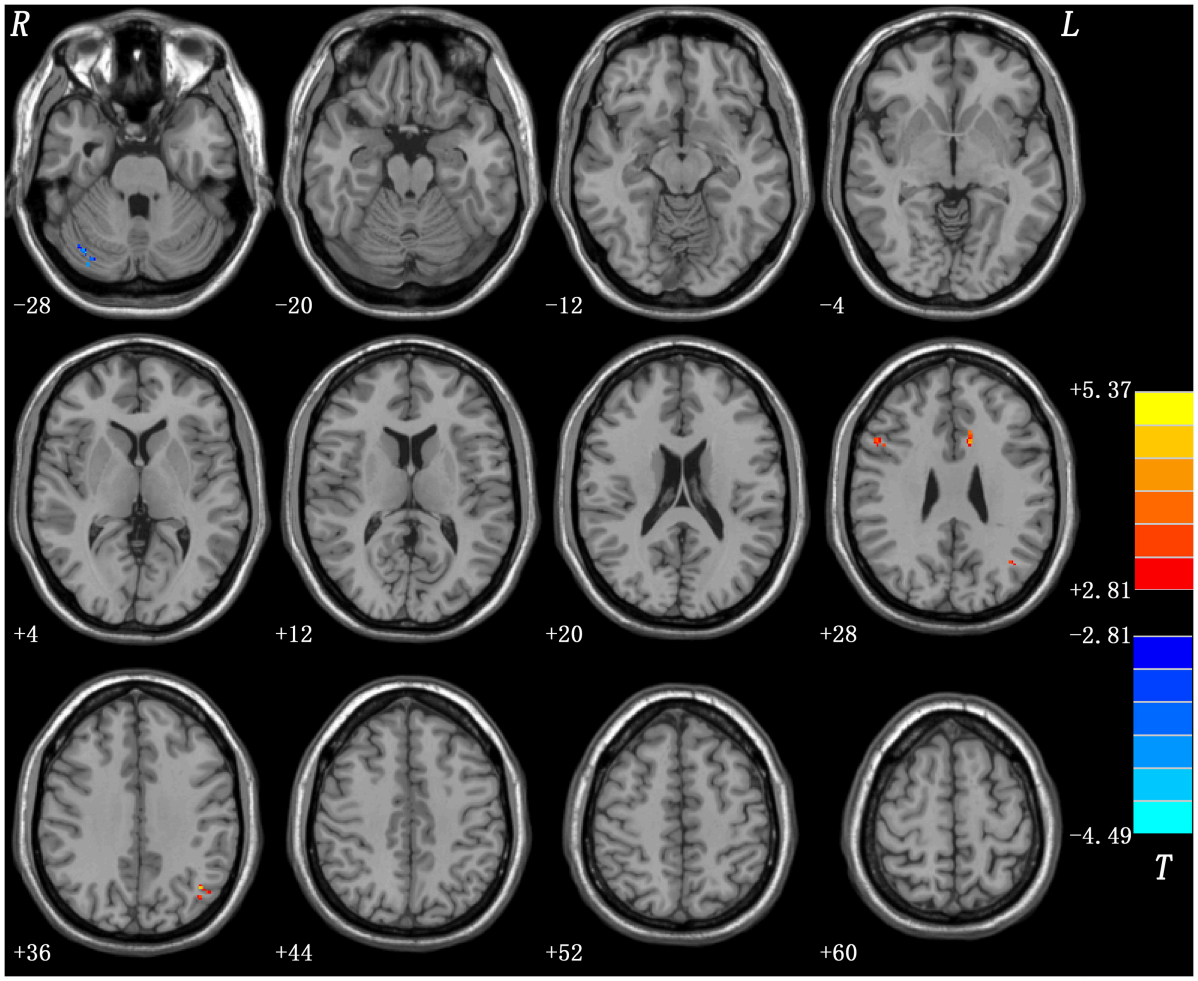
Regions with abnormal gray matter volume in the patients.

Relative to the controls, patients with SD showed significantly reduced GMV in the right cerebellum Crus I and significantly increased GMV in the left ACC, right MFG, and left AG (Table 2 and Figure 2). These four brain regions were selected as seeds for further GCA analyses.

**Table 2:** Regions with abnormal gray matter volume in the patients.

**Cluster location**	**Peak (MNI)**			**Number of voxels**	***T*-value**
	***x***	***y***	***z***		
Right Cerebellum Crus I	36	−75	−28.5	27	−3.4839
Left Anterior Cingulate Cortex	−12	18	27	35	4.7333
Right Middle Frontal Gyrus	51	15	30	24	3.9673
Left Angular Gyrus	−39	−63	33	25	4.3977

*MNI, Montreal Neurological Institute*.

### Voxel-wise GCA: seed-to-whole-brain analysis

**Figure 3:**
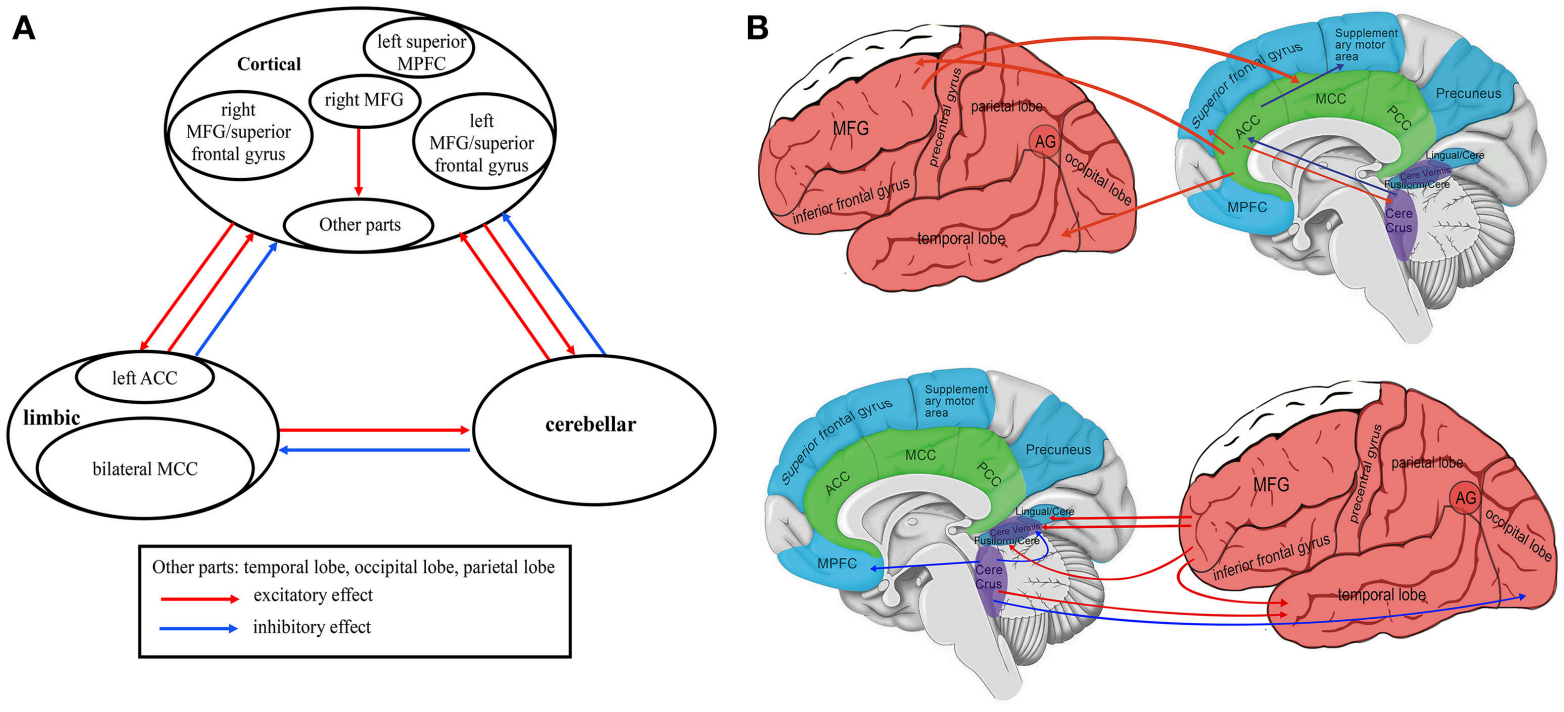
Integrated model of voxel-wise Granger causality analyses in first-episode, drug-naive somatization disorder (Seed-to-Whole-Brain Analyses): affected causal connectivities in the cortico-limbic-cerebellar circuit **(A)**. Brain regions and circuitry implicated in first-episode, drug naive somatization disorder (Seed-to-Whole-Brain Analyses) **(B)**. MFG, Middle Frontal gyrus; MPFC, Medial Prefrontal Cortex; ACC, Anterior Cingulate Cortex; MCC, Middle Cingulate Cortex; PCC, Posterior Cingulate Cortex; AG, Angular Gyrus; Cere Crus, Cerebellum Crus; Cere Vermis, Cerebellum Vermis; Lingual/Cere, Lingual Gyrus/Cerebellum Vermis; Fusiform/Cere, Fusiform Gyrus/Cerebellum. Lateral cortical regions are shown in red, medial cortical regions in blue, subcortical regions in green, and cerebellum regions in purple. The functional pathways in **(A,B)** are indicated with red arrows (excitatory effect) and blue arrows (inhibitory effect).

As shown in Table 3 and Figure 3 (Supplementary Figure S1), the patients exhibited excitatory effect from the left ACC to the left cerebellum Crus II, bilateral MFG/superior frontal gyrus, and right middle temporal gyrus (MTG) relative to the controls. In addition, the patients showed excitatory effect from the right MFG to the right fusiform gyrus/cerebellum vermis IV, V, left lingual gyrus/cerebellum vermis VI, cerebellum vermis IX, left inferior temporal gyrus, and bilateral middle cingulate cortex. Moreover, the patients exhibited inhibitory effect from the right cerebellum Crus I to the left middle occipital gyrus/inferior occipital gyrus/cerebellum VI, left superior MPFC, and left superior MPFC/ACC, from the left ACC to the left supplementary motor area.

**Table 3:** Regions with abnormal causal effect with the seeds in the patients.

**Cluster location**	**Peak (MNI)**			**Number of voxels**	***T*-value^a^**
	***x***	***y***	***z***		
**SEED-TO-WHOLE-BRAIN EFFECT**					
**Seed: Right cerebellum crus I**					
Left Middle Occipital Gyrus/Inferior Occipital Gyrus/Cerebellum VI	−45	−78	−6	235	−3.9323
Left Superior MPFC/ACC	−6	51	27	412	−4.3354
Left Superior MPFC	−9	39	51	63	−5.2241
Right Superior Temporal Gyrus	39	−39	12	34	4.1831
**Seed: Left ACC**					
Left Cerebellum Crus II	−45	−60	−45	49	3.9294
Left Middle Frontal Gyrus/Superior Frontal Gyrus	−30	45	9	59	3.8802
Right Middle Frontal Gyrus/Superior Frontal Gyrus	24	60	12	93	4.5407
Right Middle Temporal Gyrus	42	−66	15	42	4.1405
Left Supplementary Motor Area	−9	−12	57	51	−3.5602
**Seed: Right middle frontal gyrus**					
Left Inferior Temporal Gyrus	−42	9	−39	33	3.7493
Cerebellum Vermis IX	3	−51	−36	32	3.6072
Right Fusiform Gyrus/Cerebellum IV, V	21	−36	−18	120	4.2073
Left Lingual Gyrus/Cerebellum Vermis VI	−12	−39	−6	248	4.8860
Bilateral Middle Cingulate Cortex	−3	−3	33	35	3.8098
**Seed: Left angular gyrus**					
None					
**WHOLE-BRAIN-TO-SEED EFFECT**					
**Seed: Right cerebellum crus I**					
Left Middle Frontal Gyrus	−36	33	36	48	3.9856
**Seed: Left ACC**					
Left Cerebellum Crus II	−42	−63	−36	67	−4.0615
Right Middle Temporal Gyrus	39	−66	18	46	−4.1778
Right Superior Frontal Gyrus	24	63	12	38	−4.5013
**Seed: Right middle frontal gyrus**					
Left Cerebellum Crus I, II	−42	−51	−42	171	−4.225
Bilateral Lingual Gyrus/Cerebellum Vermis VI	3	−78	−24	337	−4.4932
Cerebellum IV, V	−6	−39	−9	48	−4.2817
Right Superior Parietal Lobule	24	−72	51	93	−4.2222
**Seed: Left angular gyrus**					
Left Superior Temporal Gyrus	−54	−12	6	78	4.3236
Right Inferior Frontal Gyrus	63	12	6	34	5.2976
Bilateral PCC/Precuneus	0	−15	48	174	3.6941
Right Precentral Gyrus/Postcentral Gyrus	54	−12	42	57	3.5997
Right Postcentral Gyrus	42	−33	54	37	3.9333

a *A positive/negative T value represents an increased/decreased causal effect; MNI, Montreal Neurological Institute; MPFC, Medial Prefrontal cortex; ACC, Anterior Cingulate Cortex; PCC, Posterior Cingulate Cortex*.

### Voxel-wise GCA: whole-brain-to-seed analysis

**Figure 4:**
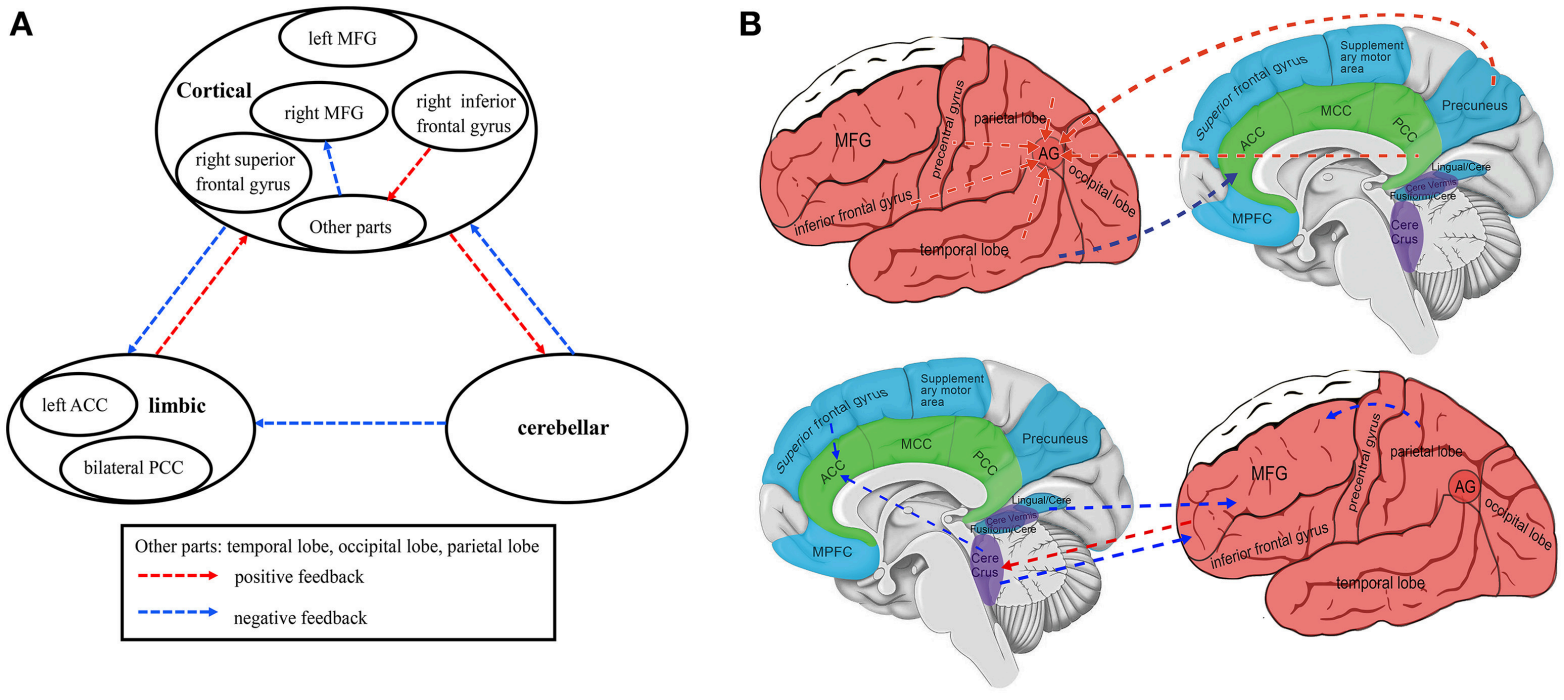
Integrated model of voxel-wise Granger causality analyses in first-episode, drug-naive somatization disorder (Whole-Brain-to-Seed Analyses): affected causal connectivities in the cortico-limbic-cerebellar circuit **(A)**. Brain regions and circuitry implicated in first-episode, drug naive somatization disorder (Whole-Brain-to-Seed Analyses) **(B)**. MFG, Middle Frontal gyrus; MPFC, Medial Prefrontal Cortex; ACC, Anterior Cingulate Cortex; MCC, Middle Cingulate Cortex; PCC, Posterior Cingulate Cortex; AG, Angular Gyrus; Cere Crus, Cerebellum Crus; Cere Vermis, Cerebellum Vermis; Lingual/Cere, Lingual Gyrus/Cerebellum Vermis; Fusiform/Cere, Fusiform Gyrus/Cerebellum. Lateral cortical regions are shown in red, medial cortical regions in blue, subcortical regions in green, and cerebellum regions in purple. The functional pathways in **(A,B)** are indicated with red dotted arrows (positive feedback) and blue dotted arrows (negative feedback).

Patients with SD showed positive feedback from the left MFG to the right cerebellum Crus I and from the left superior temporal gyrus, bilateral PCC/precuneus, right inferior frontal gyrus, right precentral gyrus/postcentral gyrus, and right postcentral gyrus to the left AG compared with healthy controls. By contrast, causal effects from the left cerebellum Crus II, right MTG, and right superior frontal gyrus to the left ACC, as well as from the left cerebellum Crus I and II, cerebellum IV, V, bilateral lingual gyrus/cerebellum vermis VI, and right superior parietal lobule to the right MFG decreased in the patients (Table 3, Figure 4 and Supplementary Figure S2).

### Correlations between anatomical alterations or causal effects and clinical variables in patients with SD

**Figure 5:**
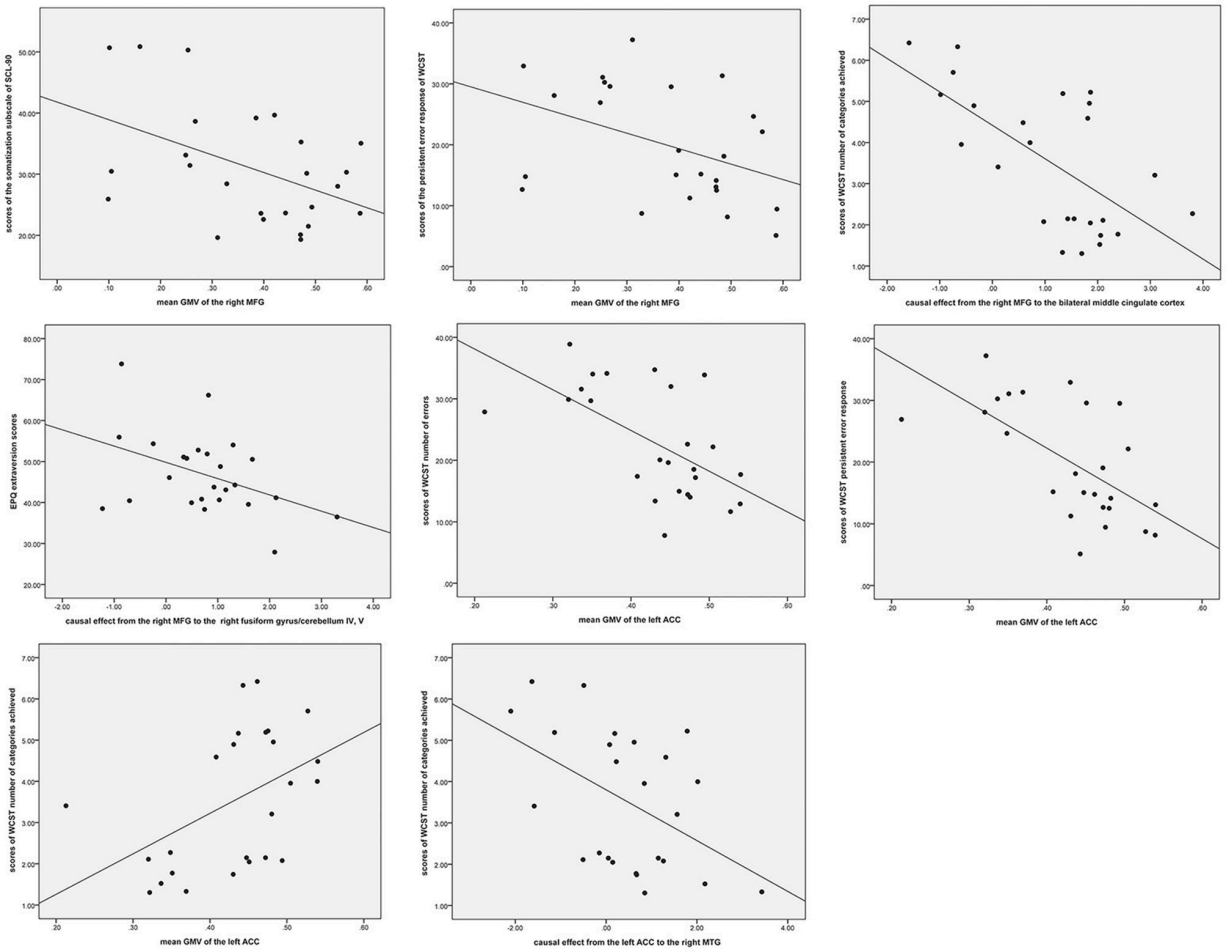
Correlations between abnormal GMV or causal effects and clinical variables in patients with somatization disorder. GMV, gray matter volume; ACC, anterior cingulate cortex; WCST, Wisconsin Card Sorting Test; WCST-Pre, persistent error response of WCST; MFG, middle frontal gyrus; MTG, middle temporal gyrus.

As shown in Figure 5, the mean GMV of the right MFG was negatively correlated with the scores of the somatization subscale of SCL-90 (*r* = −0.46, *p* = 0.027) and persistent error response of WCST (*r* = −0.415. *p* = 0.049) in the patients. Significantly negative correlations were observed between the causal effect from the right MFG to the bilateral middle cingulate cortex and scores of WCST number of categories achieved (*r* = −0.649, *p* = 0.001) and between the causal effect from the right MFG to the right fusiform gyrus/cerebellum IV and V and the EPQ extraversion scores (*r* = −0.422, *p* = 0.045). Moreover, the mean GMV of the left ACC was positively associated with the scores of WCST number of categories achieved (*r* = 0.467, *p* = 0.025) and negatively associated with the WCST number of errors and persistent error response (*r* = – 0.589, *p* = 0.003; *r* = −0.627, *p* = 0.001). Significantly negative correlation was observed between the causal effect from the left ACC to the right MTG and the scores of WCST number of categories achieved (*r* = −0.472, *p* = 0.023), and a positive correlation was found between the causal effect from the right MTG to the left ACC and scores of WCST number of categories achieved (*r* = 0.487, *p* = 0.019).

## Discussion

The current findings reveal that the causal connectivity of the cortico-limbic-cerebellar circuit is partly affected by structural alterations in patients with SD. The primary results include bidirectional cortico-limbic connectivity abnormalities, bidirectional cortico-cerebellar connectivity abnormalities, bidirectional limbic-cerebellar connectivity abnormalities, and bidirectional causal effects among the cortical regions (Figures 3, 4). Moreover, correlations between anatomical alterations or causal effects and clinical variables are observed in patients with SD, and bear clinical significance.

Gray matter deficits in the bilateral amygdala or pituitary have been reported in patients with SD by using manual volumetric analysis (20, 21). Inconsistent with these studies, the present study demonstrates that patients with SD showed increased GMV in several cortical areas, including the right MFG, left ACC, and left AG. Several factors merit consideration in interpreting the increased GMV seen in our study. First, the magnetic resonance field strength may have contributed to such increases. Previously, Atmaca et al. (20) and Yildirim et al. (21) utilized a 1.5 T magnetic resonance scanner. In the current study, we used a 3.0 T scanner, which had higher signal-to-noise ratio, better image quality, and higher resolution than the 1.5 T scanner. Second, all patients were females in the previous studies, whereas there were four male patients in our study. Sex differences in GMV may account for the inconsistency (38). Importantly, Atmaca et al. and Yildirim et al. recruited chronic and medicated patients, whereas we recruited first-episode and drug-naive patients. Therefore, the patients in our study may present with early-stage neuronal pathology at the onset of somatization symptoms.

The MFG is involved in working memory, executive functions, attention, and language skills (39–42). Increased GMV in the right MFG has not been reported in patients with SD, but increased FC in the left MFG in patients with SD has been found (43). Furthermore, negative associations were found between the GMV of the right MFG and somatization subscale of SCL-90 and persistent error response of WCST in patients with SD in this study, indicating that patients with a smaller right MFG volume experienced more severe somatic symptoms and impaired executive function. Moreover, a negative correlation was observed between the increased driving connectivity from the right MFG to the bilateral middle cingulate cortex and number of categories achieved on the WCST scores in patients, suggesting that patients with increased cortico-limbic connectivity would show prominent dysfunction in executive function. A negative correlation was also found between the EPQ extraversion scores and increased driving effect from the right MFG to the right fusiform gyrus/cerebellum IV, V in the patients, demonstrating that patients with a low EPQ extraversion (introversion) score reflect increased cortico-cerebellar connectivity. Patients who were introverts tend to be reserved, show solitary in behavior, and seek internal stimuli. Due to personality traits concentrating on internal stimuli, introverts may be more concerned about their own internal physical discomfort and be inclined to have a high risk of experiencing SD. Increased connectivity in the lobule IX-left superior MPFC with a negative correlation with the EPQ extraversion scores has been reported in patients with SD (10). In line with these findings, the present study emphasizes the importance of the cortico-limbic-cerebellar circuit in the neurophysiological mechanism of SD.

The ACC plays an important role in cognitive, emotional regulation, social evaluation, and awareness (44–47). Intriguingly, anatomical alterations of the left ACC and increased connectivity of ACC are correlated with cognitive function. First, the mean GMV of the left ACC negatively correlates with the WCST number of errors and persistent error response scores, and positively correlates with the WCST number of categories achieved scores. These correlations suggest that patients with a smaller GMV of the left ACC may display more serious cognitive impairment, such as dysfunction abstract reasoning and problem solving. ACC plays a crucial role in cognition and emotional regulation, especially in the monitoring of conflict processing (48). Our findings are in line with the results that ACC has a strong connectivity with areas involved in cognition and sensorimotor processing (49). Second, a negative correlation was observed between increased driving effect from the left ACC to the right MTG and scores of WCST number of categories achieved, corresponding to the positive correlation between decreased feedback from the right MTG to the left ACC and scores of WCST number of categories achieved. The MTG, located between the superior temporal and inferior temporal gyri, is involved in cognitive processes, such as semantic memory processing and multimodal sensory integration (50). Taken together, these findings suggest that the left ACC and increased driving effect from the left ACC to the right MTG might be an important anatomical substrate of cognitive deficits in patients with SD.

The AG plays an important role in semantic processing, language, number processing, memory retrieval, attention, reasoning, and social cognition (51, 52). A recent study suggests that patients with SD were associated with lower voxel-mirrored homotopic connectivity in the AG/supramarginal gyrus (11). Therefore, our finding of increased GVM in the AG may provide an understanding of the pathophysiology of SD.

Converging evidence suggests that the cerebellum is involved in sensorimotor control, cognitive function, and emotional control (53–56). Previous SPECT study in patients with SD found hypoperfusion in cerebellum region in four out of eleven patients with SD (14). Decreased cerebellar activity has also been found in patients with somatoform disorder during emotional empathy by using fMRI (19), suggesting that there may be important clinical significance associated with disorder-related alteration in the cerebellum. MPFC, a key node in the DMN, has an extensive connection with the affective limbic regions, including the hippocampus, amygdala, and hypothalamus. Additionally, MPFC and ACC are important parts of cortical midline structures, which play a crucial role in emotional regulation, self-referential processing, and sensory and higher-order processing. Cortical midline structures play a mediating effect in self-referential processing between sensory (sensory cortex) and advanced cognitive processing (lateral cortex). Considering many studies showing the involvement of the cerebellum in cognitive processing and emotional regulation (56–58), the present study suggests that the cerebellum is involved in the neurobiology of SD through a cerebellar-frontal connectivity.

Several limitations should be taken into consideration when interpreting the present results. First, the current study is a cross-sectional study. Longitudinal studies are needed to understand the treatment effects on the altered cortico-limbic-cerebellar circuit in SD. Second, patients with SD showed a high rate of comorbidity with depression in our study, which could have affected our findings. However, to exclude the potential effects of depression and anxiety, the HAMD and HAMA scores were used as covariates in the correlation analyses. Hence, comorbidity with depression and anxiety may have limited effects on our results. Finally, the sample size was relatively small. A large sample size is needed to confirm or refute the current results.

Despite these limitations, this study is the first to explore the causal connectivity affected by structural alterations in patients with SD at rest. These findings demonstrate the partial effects of structural alteration on the cortico-limbic-cerebellar circuit in first-episode, drug-naive patients with SD. Correlations are observed between anatomical alterations or causal effects and clinical variables in patients with SD, and bear clinical significance. The present study emphasizes the importance of the cortico-limbic-cerebellar circuit in the neurobiology of SD.

## Author contributions

RL, FL, QS, ZZ, JZ, YW, RW, JZ, and WG: acquired the data, designed the study, contributed to data analysis, interpretation of data and wrote the article. All authors reviewed and gave final approval of publication.

### Conflict of interest statement

The authors declare that the research was conducted in the absence of any commercial or financial relationships that could be construed as a potential conflict of interest. The reviewer XX and handling Editor declared their shared affiliation.

## Supplementary material

Supplementary Figure S1 Voxel-wise Granger causality analyses in patients with somatization disorder: Seed-to-Whole-Brain Analyses.

Supplementary Figure S2 Voxel-wise Granger causality analyses in patients with somatization disorder: Whole-Brain-to-Seed Analyses.
