# Supplementary figures and images for: Bidirectional Causal Connectivity in the Cortico-Limbic-Cerebellar Circuit Related to Structural Alterations in First-Episode, Drug-Naive Somatization Disorder
Source: Front Psychiatry. 2018 Apr 26;9:162. doi: 10.3389/fpsyt.2018.00162 (PMC5932337; doi:10.3389/fpsyt.2018.00162)

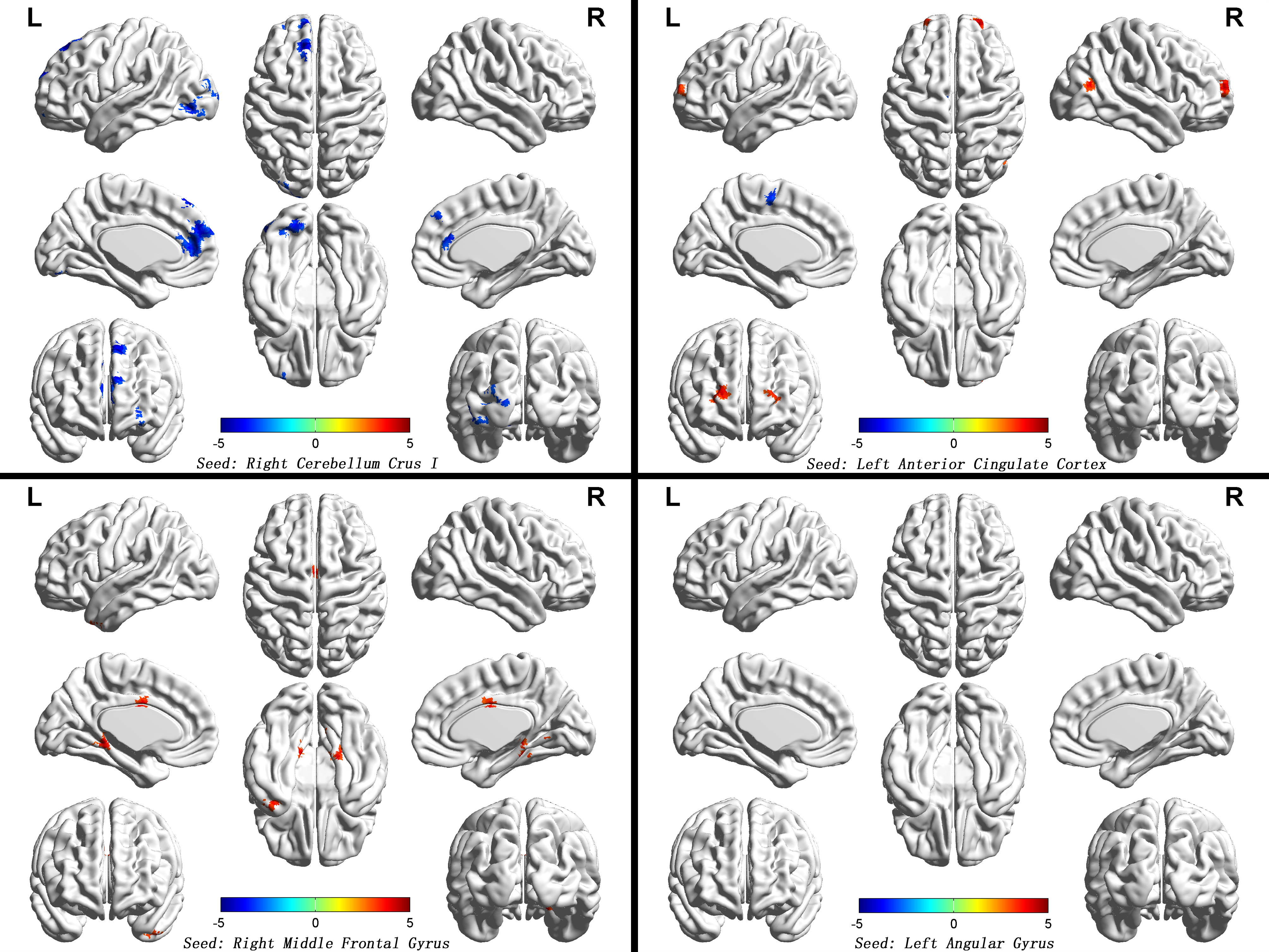

Supplement: Supplementary Figure S1 — Voxel-wise Granger causality analyses in patients with somatization disorder: Seed-to-Whole-Brain Analyses. [file Image_1.TIF]

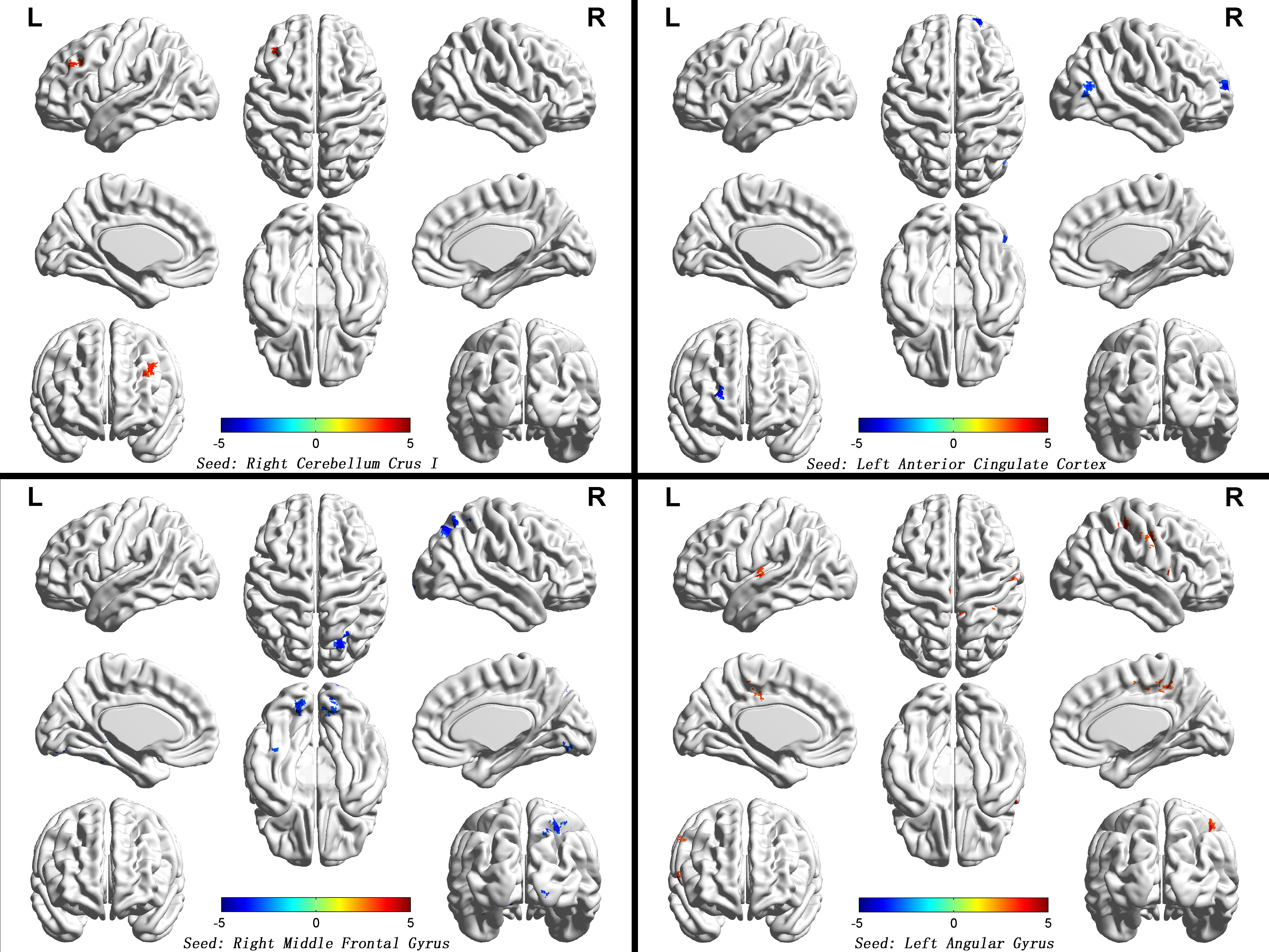

Supplement: Supplementary Figure S2 — Voxel-wise Granger causality analyses in patients with somatization disorder: Whole-Brain-to-Seed Analyses. [file Image_2.TIF]
