# Supplementary material for: Bidirectional Causal Connectivity in the Cortico-Limbic-Cerebellar Circuit Related to Structural Alterations in First-Episode, Drug-Naive Somatization Disorder
Source: Front Psychiatry. 2018 Apr 26;9:162. doi: 10.3389/fpsyt.2018.00162 (PMC5932337; doi:10.3389/fpsyt.2018.00162)
Supplement: Supplementary file 3 [file Presentation_1.PDF]

Title:

Bidirectional causal connectivity in the cortico-limbic-cerebellar circuit related to structural alterations in first-episode, drug-naive somatization disorder

Authors:

Ranran Li [a], Feng Liu[b], Qinji Su[c], Zhikun Zhang [c], Jin Zhao[a], Ying Wang [a], Renrong Wu[a], Jingping Zhao [a], Wenbin Guo [a*]*

Affiliation/address:

a. Department of Psychiatry, the Second Xiangya Hospital of Central South University. Changsha, Hunan, China.
b. Department of Radiology, Tianjin Medical University General Hospital, Tianjin, China.
c. Mental Health Center of the First Affiliated Hospital, Guangxi Medical University, Nanning, Guangxi, China.

Corresponding authors:

Jingping Zhao

Department of Psychiatry, the Second Xiangya Hospital of Central South University, Changsha, Hunan 410011, China.

E-mail: zhaojingping@csu.edu.cn

Wenbin Guo

Department of Psychiatry, the Second Xiangya Hospital of Central South University, Changsha, Hunan 410011, China.

E-mail: guowenbin76@csu.edu.cn

Tel.: +86 731 85360921

1.2. MRI Acquisition and Functional Data Preprocessing

Whole-brain imaging was acquired on a 3.0 T Siemens scanner. Subjects were instructed to relax with their eyes closed and keep awake. High-resolution volumetric data were acquired using a spoiled gradient recall sequence (repetition time = 2300 ms, echo time = 2.98 ms,, inversion time = 900 ms, flip angle = 9°, acquisition matrix = 256 × 256, field of view = 256 × 256 mm^3, slice thickness = 1 mm, no gap, and 176 slices),

and resting-state functional images were obtained adopting a gradient-echo echo-planar imaging sequence (repetition time = 2000 ms, echo time = 30 ms, 30 slices, matrix= 64 × 64, flip angle = 90°, field of view= 24 cm, 0.4 mm gap, 4 mm slice thickness, and 250 volumes lasting for 500 s).

Functional data were preprocessed with the software DPABI in Matlab (32). After head movement and slice timing correction, subjects with a maximal translation of x, y, or z more than 2 mm or a maximal rotation more than 2° were excluded. The obtained images were spatially normalized to the Montreal Neurological Institute (MNI) echo-planar imaging template in SPM8 (spatial resolution = 3 × 3 × 3 mm ^{3}). The time series was then bandpass filtered (0.01-0.08 Hz) and linearly detrended. Several spurious covariates along with their temporal derivatives containing six head motion parameters obtained by rigid body correction, signal from a ventricular ROI, and the effect of the signal from white matter region were removed.

Reference

32. Yan, C.G., Wang, X.D., Zuo, X.N., and Zang, Y.F. DPABI: Data Processing & Analysis for (Resting-State) Brain Imaging. *Neuroinformatics* (2016)14 (3): 339-51. doi: 10.1007/s12021-016-9299-4.
